# Plasma Proteome Fingerprints Reveal Distinctiveness and Clinical Outcome of SARS-CoV-2 Infection

**DOI:** 10.3390/v13122456

**Published:** 2021-12-07

**Authors:** Wolfgang Bauer, Marcus Weber, Eva Diehl-Wiesenecker, Noa Galtung, Monika Prpic, Rajan Somasundaram, Rudolf Tauber, Jochen M. Schwenk, Patrick Micke, Kai Kappert

**Affiliations:** 1 Department of Emergency Medicine, Charité—Universitätsmedizin Berlin, Corporate Member of Freie Universität Berlin and Humboldt-Universität zu Berlin, Hindenburgdamm 30, 12203 Berlin, Germany; wolfgang.bauer@charite.de (W.B.); eva.diehl-wiesenecker@charite.de (E.D.-W.); noa.galtung@charite.de (N.G.); rajan.somasundaram@charite.de (R.S.); 2 Zuse Institute Berlin (ZIB), Takustraße 7, 14195 Berlin, Germany; weber@zib.de; 3 Institute of Laboratory Medicine, Clinical Chemistry and Pathobiochemistry, Charité—Universitätsmedizin Berlin, Corporate Member of Freie Universität Berlin and Humboldt-Universität zu Berlin, Augustenburger Platz 1, 13353 Berlin, Germany; monika.prpic@charite.de (M.P.); rudolf.tauber@charite.de (R.T.); 4 Labor Berlin—Charité Vivantes GmbH, 13353 Berlin, Germany; 5 Science for Life Laboratory, KTH-Royal Institute of Technology, Tomtebodavägen 23, 17165 Solna, Sweden; jochen.schwenk@scilifelab.se; 6 Department of Immunology, Genetics and Pathology, Uppsala University, Dag Hammarskjölds Väg 20, 75185 Uppsala, Sweden; patrick.micke@igp.uu.se

**Keywords:** COVID-19, proteomics, risk prediction, proximity extension assay, emergency medicine

## Abstract

Background: We evaluated how plasma proteomic signatures in patients with suspected COVID-19 can unravel the pathophysiology, and determine kinetics and clinical outcome of the infection. Methods: Plasma samples from patients presenting to the emergency department (ED) with symptoms of COVID-19 were stratified into: (1) patients with suspected COVID-19 that was not confirmed (*n* = 44); (2) non-hospitalized patients with confirmed COVID-19 (*n* = 44); (3) hospitalized patients with confirmed COVID-19 (*n* = 53) with variable outcome; and (4) patients presenting to the ED with minor diseases unrelated to SARS-CoV-2 infection (*n* = 20). Besides standard of care diagnostics, 177 circulating proteins related to inflammation and cardiovascular disease were analyzed using proximity extension assay (PEA, Olink) technology. Results: Comparative proteome analysis revealed 14 distinct proteins as highly associated with SARS-CoV-2 infection and 12 proteins with subsequent hospitalization (*p* < 0.001). ADM, IL-6, MCP-3, TRAIL-R2, and PD-L1 were each predictive for death (AUROC curve 0.80–0.87). The consistent increase of these markers, from hospital admission to intensive care and fatality, supported the concept that these proteins are of major clinical relevance. Conclusions: We identified distinct plasma proteins linked to the presence and course of COVID-19. These plasma proteomic findings may translate to a protein fingerprint, helping to assist clinical management decisions.

## 1. Introduction

SARS-CoV-2 has become a pandemic, affecting more than 219 million people (October 2021), afflicting a significant percentage of infected individuals with symptoms severe enough to require hospitalization. The treatment of COVID-19 patients is challenging because of the clinical variability of the disease, ranging from asymptomatic to severe courses, with potentially fatal outcomes [1,2,3,4]. Several biomarkers and protein signatures have been suggested that can predict the course of COVID-19, particularly a severe clinical outcome, e.g., intensive care unit (ICU) treatment, mechanical ventilation (MV), and multi organ failure [5,6,7,8,9].

Well-established clinical chemistry enzymes and inflammatory markers for risk assessment of COVID-19 patients mostly present the current standard of care in clinical settings [10]. In particular, patterns of markers reflecting inflammation, organ dysfunction, dysregulated coagulation, as well as changes in immune cells were suggested to improve monitoring and prediction of the course of the disease. However, most of these studies were designed to classify the severity of COVID-19 rather than to characterize the pathophysiology of SARS-CoV-2 infection [11]. Parameters, such as decreased platelet count, elevated levels of D-dimer, C-reactive protein (CRP), interleukins (IL), ferritin, and troponin were identified as risk factors for severe COVID-19, yet—as known predictors of severe infections and sepsis—lack specificity [12].

Recently, more advanced techniques, using proteomic, metabolomic, complex flow cytometry, as well as transcriptome analyses, and even single cell sequencing were used to describe COVID-19 disease progression [13,14,15,16,17]. “Omic” technologies by which proteomes and biological signatures can be described in a largely unbiased manner are not yet well established in the hospital environment and in the clinical laboratory. Moreover, these techniques lack standardization, robustness, and the sensitivities for protein detection are hitherto poor, preventing clinical applicability.

SARS-CoV-2 infection triggers a potent immune response [18,19]. A better understanding of these mechanisms and the proteins involved enables understanding of the specific pathophysiology of COVID-19 and suggests targeted therapeutic approaches.

The aim of our study was to identify pathognomonic protein signatures of COVID-19 related to inflammation and cardiometabolic processes by multiplexed and ultra-sensitive proteomic assays [20]. We further included patients with clinically suspected COVID-19 who were subsequently ruled-out for SARS-CoV-2 infection, but presented with COVID-19 comparable symptoms to the emergency department (ED). Thus, patients with suspected COVID-19 in the ED were analyzed in-depth for the circulating proteomes to determine (i) the pathophysiology of COVID-19; (ii) clinical outcome compared to standard of care diagnostics; and (iii) the kinetics of the proteome impacting disease development.

## 2. Materials and Methods

### 2.1. Study Population

We performed a non-interventional, observational study at the university hospital Charité—Universitätsmedizin Berlin. The study was approved by the ethics committee at Charité—Universitätsmedizin Berlin, Campus Virchow-Klinikum, Germany (no. EA2/095/20). All experiments were performed in accordance with relevant guidelines and regulations. From 19 March, 2020 to 18 June, 2020, we enrolled a consecutive sample of 141 patients presenting to the emergency department with clinically suspected COVID-19. Patients were tested by SARS-CoV-2 polymerase chain reaction (PCR) in pharyngeal swabs. The PCR results, which were received within 48 h, did not affect treatment at presentation. Amongst the 141 patients, 97 were diagnosed with COVID-19 of which 53 were hospitalized and 44 were discharged to home care. A total of 44 patients were tested negative for SARS-CoV-2. Additionally, 20 patients with negative SARS-CoV-2 PCR swabs and no clinical suspicion of COVID-19 (tested as general care within their admission to the emergency department, due to, e.g., minor traumata) were considered a reference cohort.

All patients enrolled were clinically examined, diagnosed, and treated to the standard of care, including blood samples for laboratory blood cell count and differentiation, hemostaseological and clinical chemistry analyses. We monitored consecutive disease progression, categorized by ICU admission vs. non-ICU treatment, requirement for MV, thromboembolic events (TE), discharge, and death.

### 2.2. Standard of Care Blood Analyses

Lithium heparin plasma, EDTA plasma, citrate plasma (3.2% citrate), as well as serum were isolated after centrifugation (2200× *g* for 10 min; all tubes were from Greiner, Bio-One, Kremsmünster, Austria). Routine laboratory values were measured as standard of care at the time of admission to the ED or consecutively during the hospital stay. Hematology was measured on a Sysmex hematology analyzer within an XN-2000 configuration in EDTA tubes, clinical chemistry parameters were determined on Roche cobas c701, e602, and e801 analyzers within a cobas 6000/8000 configuration in heparin plasma or serum. Coagulation testing was performed using citrate plasma and a STAR MAX analyzer (Stago Germany GmbH, Düsseldorf, Germany).

### 2.3. Fingerprinting the Circulating Proteome

To measure the circulating proteome, aliquots of standard of care samples were used that were taken from patients at presentation to the ED. In cases of hospitalization (*n* = 53), additional samples were collected from the latest time a blood sample was taken (either prior to discharge or death) and at the mean of hospital stay (total of 3 samples). For 10 cases, the hospital stay was covered by a total of 7 plasma samples each taken at 7 consecutive times.

Plasma levels of altogether 177 different proteins were analyzed using Olink’s panels Cardiovascular II (Lot #B02623) and Inflammation (Lot #B02608) that employ their proximity extension assay (PEA) technology [20]. Briefly, one microliter of plasma was incubated with a set of probes, each consisting of an antibody conjugated to a specific DNA oligonucleotide. Once a protein is recognized by a pair of probes, the DNA oligonucleotides of the antibody pairs, now in close proximity, are allowed to hybridize to each other and are extended by enzymatic polymerization. The newly formed DNA molecule is then amplified and quantified by real-time PCR. The PCR results were analyzed as normalized protein expression (NPX) values on a log2 scale. NPX values were obtained by normalizing Cq-values against extension controls, inter-plate control, and a correction factor. A high NPX value corresponds to a high protein concentration and expresses relative quantification between samples but represents no absolute quantification that would allow comparing the levels of different proteins to another. Results from the proteome analysis did not impact the treatment of patients.

### 2.4. Statistics

Identifying relevant parameters. The aim was to identify proteins that indicated differences with regard to patient classification before hospitalization and to clinical endpoints. For every parameter, measured values were binned into “low”, “middle”, “high” with the same population size for each quantile and two half-populated quantiles were added for the upper and lower outliers. The two rows (comparing two classes of patients) of the corresponding cross table should overlap as little as possible, determined by the chi^2^-independence test of those cross tables. A classification was defined as “likely not independent from the measured parameter”, if the *p*-value was <0.001, approximately one fifth of 1/184 (total number of parameters including analytes spotted on both PEA-plates).

Restricting to clinically relevant parameters. The quantized chi^2^-test led to a set of identified parameters. To reduce the risk that correlations between parameter quantile and clinical events were due to overfitting, the data set was further restricted to parameters which correlated to at least one cohort assignment (COVID-19 yes/no, hospitalization yes/no) as well as to at least one of the clinical endpoints (ICU admission, requirement for MV, TE, death).

Principal component analysis (PCA). Based on the matrix M of the selected clinically relevant parameters versus the first measurements of hospitalized COVID-19 patients (*n* = 53), a singular value decomposition (PCA) was performed with M = *U^T^*∑V.

The prognostic and diagnostic strength of the circulating proteins was compared using area under the receiver operating characteristics (AUROC), calculated with the R package pROC. Standard of care parameters and baseline characteristics were compared using Mann–Whitney U and Fisher’s exact tests.

All statistical analyses were performed on R version 3.6.1 and MATLAB/Octave.

## 3. Results

### 3.1. Patient Stratification, Clinical and Standard of Care Laboratory Assessment

**Figure 1:**
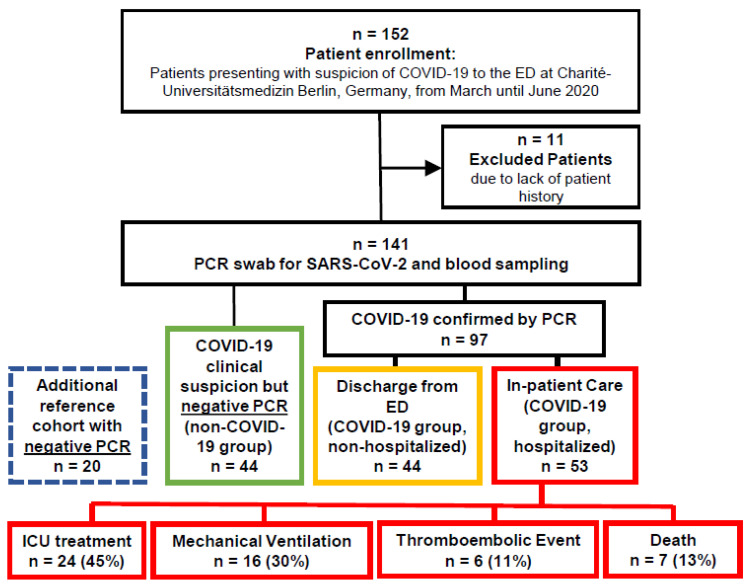
Study outline.

Altogether, *n* = 141 patients at the ED were enrolled and allocated to the following major subgroups (Figure 1): (1) patients with negative SARS-CoV-2 PCR results but with clinical symptoms compatible with COVID-19 (COVID-19 negative group, *n* = 44); (2) patients with positive SARS-CoV-2 PCR results but without hospitalization (non-hospitalized COVID-19 group, *n* = 44); (3) patients with positive SARS-CoV-2 PCR result and hospitalization (hospitalized COVID-19 group, *n* = 53). Within the hospitalized COVID-19 group, 24 patients were admitted to the ICU, 16 with indication for MV ventilation, 6 experienced TE, and 7 patients died. Baseline demographics for all patient groups are shown in Supplemental Table S1. The groups did not show any clinically significant or unexpected differences with regard to sex and major comorbidities.

**Figure 2:**
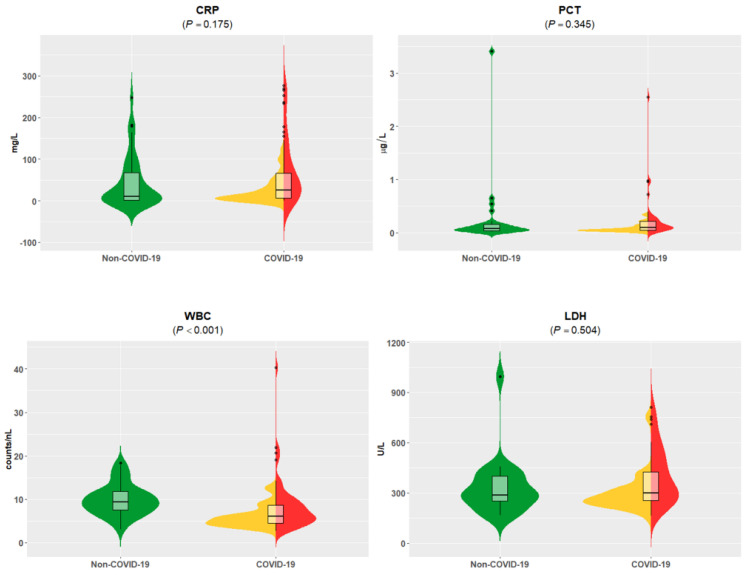
Standard of care parameter-values of C-reactive protein (CRP), procalcitonin (PCT), white blood cell (WBC) counts, and lactate dehydrogenase (LDH) of combined groups without COVID-19 were compared to COVID-19 hospitalized and non-hospitalized patients. Data are visualized by violin plots, which are a combination of kernel density plots, and box plots depicting median, 25th, and 75th percentiles. No significant differences were detectable for CRP, PCT, and LDH. Units are as follows: CRP (mg/L), PCT (µg/L), WBC (cells/nL), LDH (U/L). Significantly lower WBC levels were found in patients with confirmed SARS-CoV-2 infection compared to non-COVID-19 patients (*p* < 0.001). Green = non-COVID-19; yellow = COVID-19 without hospitalization; red = COVID-19 with hospitalization; black dots: outliers.

At admission to the emergency department, the initial clinical laboratory data revealed no major differences in clinical chemistry parameters when symptomatic non-COVID-19 patients were compared with combined hospitalized and non-hospitalized COVID-19 patients (Supplemental Table S2). Only white blood cell (WBC) counts were lower in COVID-19 patients compared to non-COVID-19 patients. Notably, the standard of care parameters CRP, procalcitonin (PCT), and lactate dehydrogenase (LDH) did not differ, although they were frequently reported as COVID-19-associated analytes [12]. Thus, these analytes did not present a pathognomonic COVID-19 pattern for patients in the ED (Figure 2). As expected, our comparison of hospitalized and non-hospitalized COVID-19 patients found significantly higher LDH, CRP, PCT, and troponin T (TNT) concentrations and lower estimated glomerular filtration rates (eGFRs) among the COVID-19 inpatients (Supplemental Table S2) [12].

### 3.2. Plasma Proteins Associated to COVID-19 Status

**Figure 3:**
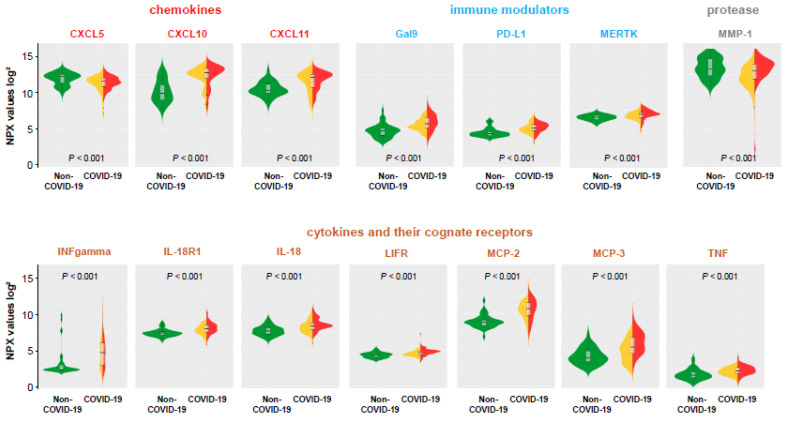
Proteome analyses—PEA values of discriminatory analytes with significant differences (*p* < 0.001) between non-COVID-19 and COVID-19 patients. Data are visualized by violin plots, which are a combination of kernel density plots, and box plots depicting median, 25th, and 75th percentiles. A high-normalized protein expression (NPX) value resembles a high protein concentration and expresses the relative quantification between samples. NPX values are given on a log-scale. Green = non-COVID-19; yellow = COVID-19 without hospitalization; red = COVID-19 with hospitalization; black dots: outliers.

The levels of 177 individual circulating proteins were determined in the four study groups at the time of admission (Supplemental Table S3, including full names and abbreviations of analyzed proteins). As highlighted in Figure 3, within this proteome analysis, there were only 14 mainly inflammatory proteins (CXCL10, CXCL11, CXCL5, Gal-9, INF-gamma, IL-18, IL-18R1, LIF-R, MCP-2, MCP-3, MERTK, MMP-1, PD-L1, and TNF) that differed significantly (*p* < 0.001) between COVID-19 patients (hospitalized and non-hospitalized) compared to those with a symptomatic non-COVID-19 diagnosis. This suggests pathognomonic proteome changes triggered by SARS-CoV-2, but also highlights that only 8% were changed in the cross-sectional analysis when compared to other symptomatic patients (Table 1). The assumption that these proteins define a pathophysiological signature of COVID-19 was underlined by the finding that no significant differences of protein levels were detected for all different 177 proteins analyzed between the reference subgroup and the symptomatic non-COVID-19 groups (significance level of *p* < 0.001, Supplemental Table S3).

### 3.3. Comparison of Hospitalized and Non-Hospitalized COVID-19 Patients

**Figure 4:**
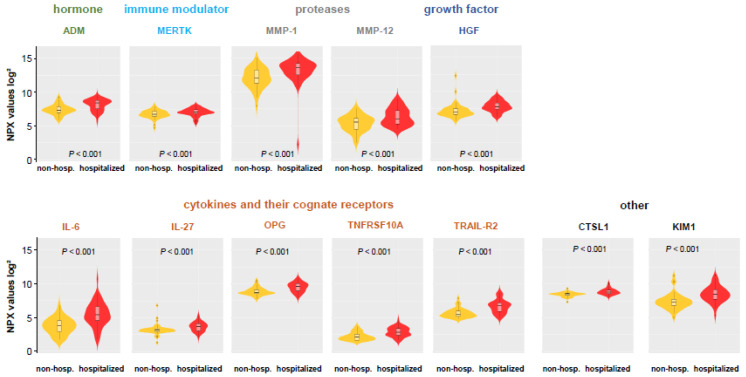
Proteome analyses—PEA values of discriminatory analytes within COVID-19 group, either non-hospitalized (outpatients) or hospitalized (inpatients). Data are visualized by violin plots, which are a combination of kernel density plots, and box plots depicting median, 25th and 75th percentiles. A high-normalized protein expression (NPX) value resembles a high protein concentration and expresses the relative quantification between samples. NPX values are given on a log-scale. Yellow = COVID-19 without hospitalization; red = COVID-19 with hospitalization; black dots: outliers.

Next, we focused on protein levels with regard to differences between COVID-19 patients that were discharged from the ED based on clinical evaluation (non-hospitalized COVID-19 group) or were subsequently hospitalized (hospitalized COVID-19 group). We identified a subset of protein analytes that were significantly (*p* < 0.001) elevated in hospitalized COVID-19 patients (ADM, CTSL1, HGF, IL-27, IL-6, KIM1, MERTK, MMP-1, MMP-12, OPG, TNFRSF10A, and TRAIL-R2) (Figure 4) (medians, interquartile ranges, and *p*-values are shown in Supplemental Table S3). The shortlist proteins include known markers of cellular degradation, hormones, as well as interaction with microbes and viruses.

### 3.4. Proteome Analyses in Hospitalized COVID-19 Patients with Regard to Clinical Outcome

Using samples collected at the initial admission to the ED, we investigated the associations of the circulating proteins with outcomes in hospitalized COVID-19 patients represented by four clinical events: ICU treatment, need for mechanical ventilation (MV), TE, and death (Table 2). Only the plasma levels of CCL23, IL6, MCP-1, MCP-3, PD-L1, and TRAIL-R2 were significantly higher (*p* < 0.001) in patients who subsequently received ICU treatment. Furthermore, a subset of five proteins (DCN, IL6, MCP-1, MCP-3, and TRAIL-R29) was significantly higher (*p* < 0.001) in patients with subsequent MV. Interestingly, the COVID-19 specific elevation of MCP-3, predicted, in addition to ICU care also the need for ventilation. Finally, the protein levels of ADM (*p* < 0.0001) and LPL (<0.0002) were strongly associated with fatal outcome.

Interquartile ranges for protein levels as well as standard of care parameters CRP, PCT, WBC, and LDH with regard to clinical outcome are shown in Table 2 and AUROC values in Supplemental Table S4. A number of analytes had high AUROCs with respect to fatal outcome, in particular ADM (0.87), CXCL10 (0.82), CXCL11 (0.82), DCN (0.80), IL-27 (0.82), and TNF (0.80). Noteworthy, no standard of care parameter (CRP, PCT, WBC, and LDH) was significantly accurate for the analyzed outcomes.

### 3.5. A Proteomic Fingerprint Predicts Outcome

**Figure 5:**
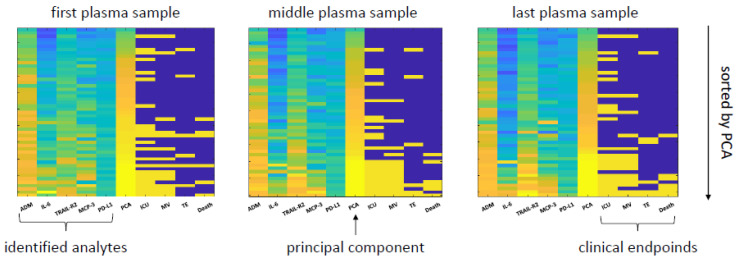
Heat map of predictive performance—the heat map presents the relative values of the five selected analytes (ADM, IL-6, MCP-3, TRAIL-R2, PD-L1) and the corresponding principal component analyses (PCA, as a combination of these markers) in relation to four clinical events (ICU treatment, TE, MV, and death). The first heat map (left) presents values based on plasma taken initially in the ED. The middle heat map presents values from the plasma sample at the middle of the hospital stay, and the last heat map plasma sample from the latest time point (either prior to discharge of the patient or before fatal outcome). The samples are sorted according to the value of PCA.

To further increase the predictive power of the plasma proteomics, we combined the different individual proteins into a single model. Based on our mathematical approach to identify relevant parameters, we selected five patient-discriminatory proteins (ADM, IL-6, MCP-3, TRAIL-R2, PD-L1) that were predictive for COVID-19 rule-in and/or hospitalization and at least for one of the following events: ICU treatment, TE, MV, and death. The principal component analysis (PCA) of these proteins (ADM, IL-6, MCP-3, TRAIL-R2, PD-L1) is shown in Figure 5.

**Figure 6:**
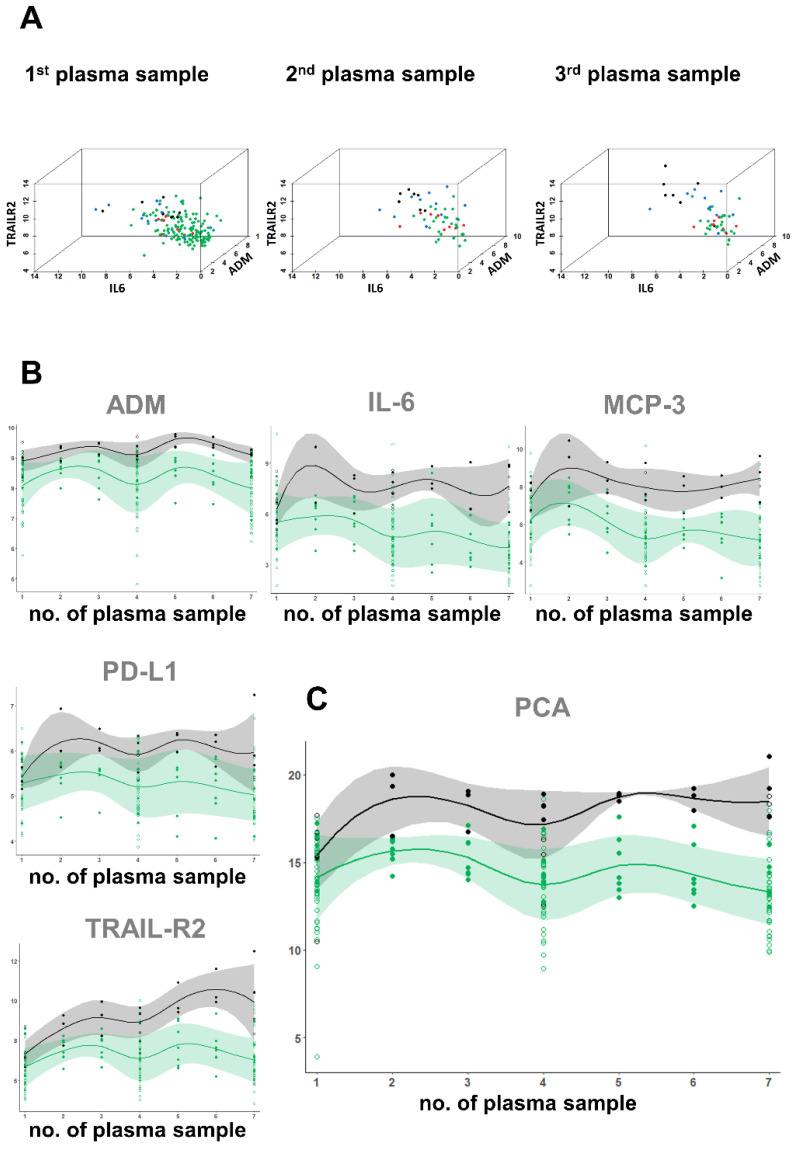
Illustration of predictive protein values and kinetics as well biological variation of proteins during hospital stay—(**A**) three-dimensional illustration of predictive protein values. Green dots: non-hospitalized COVID-19; red dots: hospitalized COVID-19 with ICU admission; blue dots: hospitalized COVID-19 with ICU admission and requirement for mechanical ventilation; Black dots: COVID-19 with fatal outcome. (**B**) Kinetics of the five selected markers separately (ADM, IL-6, MCP-3, PD-L1, TRAIL-R2) and (**C**) the PCA based on the combination of the analytes. The *x*-axis represents seven different time points of plasma procurement, with the first (1) taken at the ED and the seventh (7) isolated prior to discharge or death of the patient. Samples two to six were isolated during the hospital stay at approximately similar time intervals. Depicted are values of all *n* = 53 hospitalized COVID-19 patients with three time points. For *n* = 10 (solid dots) patients plasma samples at seven time points were analyzed. Open dots represent patients with three time points. Green dots = discharged patients, black dots = patients with fatal outcome. Shown are data for individual COVID-19 patients with survival (solid green dots = patients with seven time points, open green dots = patients with three time points) and mean values (solid green line) with standard deviation (light green area) and data for individual COVID-19 patients with fatal outcome (solid black dots = patients with seven time points, open black dots = patients with three time points) and mean values (solid black lines) with standard deviation (grey area).

To illustrate the proteome-based clustering of patient groups in terms of clinically relevant events, we selected those three proteome parameters from the identified proteins, which predicted hospitalization (ADM, IL-6, TRAIL-R2). We plotted this projected plasma proteomics data into a three-dimensional space (Figure 6A). For the analysis using ED samples only, we included all COVID-19 patients (non-hospitalized and hospitalized, *n* = 97). For the middle and last sampling time point, we only projected the data from the hospitalized patients. The scatter plots reveal a robust clustering of the different clinical groups and even more distinct separation during the course of COVID-19 infection.

Similarly, the high-resolution kinetic (Figure 6B) of the selected markers ADM, IL-6, MCP-3, TRAIL-R2, and PD-L1 demonstrated prognostic value from ED blood samples. The relative levels of the individual markers fluctuated slightly during hospitalization, a disadvantage for diagnostics, with the exception of TRAIL-R2, which increased in nearly all of patients with fatal outcome during hospital stay. However, by combining these markers into a panel using PCA, the compound fingerprint remained stable over the hospitalization time (Figure 6C) and was clearly not dependent of medical intervention (ICU, MV, and medication). The data that support the findings of this study are available on request from the corresponding author. A correlation matrix between the selected markers and the standard of care infection markers WBC, CRP, and PCT is shown in Supplemental Figure S1.

## 4. Discussion

Our in-depth and targeted analysis of the immune and cardio-metabolism related plasma proteins described the molecular phenotypes related to a specific pathophysiology of COVID-19 and outlined that circulating proteins can predict clinical outcome. The high predictive accuracy, in particular with regard to fatal outcome, together with stable protein kinetics provide a strong rationale to further evaluate the identified proteins for clinical diagnostics. Moreover, we present a composite risk fingerprint that robustly predicts COVID-19 mortality in patients at initial presentation in the ED. Our study confirms previously suggested immune-markers, such as TNF and IL-6 [21,22], as well as hitherto unrecognized proteins as novel indicators for further disease progression.

### 4.1. How Can Proteomic Fingerprinting Help Clinicians Better Understand COVID-19?

Organ failure in severe COVID-19 cases seems to be due to this excessive reaction, often described as a cytokine storm, rather than due to viral virulence [23,24,25]. Routinely measured markers, such as hematological, hemostaseological and clinical chemistry parameters, were extensively evaluated [24,26]. However, while some provide prognostic information, their lack of specificity for COVID-19 pathophysiology and their poor performance for long-term clinical outcome limits their clinical value.

### 4.2. What Are the Advantages of Our Analysis Design?

To gain a greater overview we included not only patients with suspected COVID-19 and proven SARS-CoV-2 infection in pharyngeal swabs, but also patients with COVID-19 typical symptoms, but negative SARS-CoV-2 PCR (symptomatic COVID-19 negative group). COVID-19 positive patients were further subdivided into groups requiring hospitalization or not. In the COVID-19 positive and hospitalized group, we isolated three and up to seven longitudinal samples per patient in the hospitalization follow-up. By comparing non-COVID-19 and COVID-19 patients presenting with similar symptoms to the ED, the study design has the advantage of describing proteomic aspects of the pathophysiology of COVID-19, i.e., to identify proteins and proteome changes specific to COVID-19 patients and not for a respiratory symptom pattern. In the COVID-19 patient group, protein markers can further be evaluated in predictive models, either as early time point markers or in protein kinetics during disease course.

### 4.3. What Distinguishes Patients with SARS-CoV-2 Infection from Patients with Similar Symptoms but Negative PCR Results?

The broad and sensitive proteomic analyses identified a subset of chemokines (CXCL5, CXCL10, CXCL11), immune modulators (Gal-9, PD-L1, MERTK), cytokines and their cognate receptors (IFN-gamma, IL-18, IL-18R1, LIF-R, MCP-2, MCP-3, TNF), and proteases (MMP-1), as being significantly elevated in COVID-19 patients compared to non-COVID-19 patients with similar symptoms.

SARS-CoV-2 infection rapidly triggers the release of acute phase reactants, such as INF gamma and interleukins, which is considered a general characteristic of COVID-19 [27]. Indeed, some of these pathognomonic parameters have previously been shown to be associated with SARS-CoV-2 at some point between initial infection and clinical deterioration [23,24,28].

Interestingly, only few of the COVID-19 plasma markers are also related to disease outcome. This indicates that immune activation is a feature of symptomatic COVID-19 rather than a sign for deterioration. Thus, elevated cytokine and interleukin levels, including IL-2, IFN gamma, and TNF described in several studies [24,28] are pathognomonic for SARS-CoV-2 infection in general, but seem not *primarily* related to the feared and potentially lethal hyperactivation of the immune system.

Nevertheless, to draw conclusions of the immunological essence of COVID-19 is difficult and might not only depend on the phase of infection, but also be influenced by the individual host response, as well as host-dependent co-factors, such as age, comorbidities, and coinfections.

### 4.4. Which Plasma Proteins Indicate a Severe Outcome in COVID-19 Patients?

Our model identified 16 markers that were associated with high significance (<0.001) with clinical events (hospitalization, mechanic ventilation, thromboembolic event, or death). Remarkably, only nine markers (ADM, CCL23, DCN, IL6, LPL, MCP1, MCP3, PD-L1, and TRAIL-R2) were associated with clinical events after hospitalization, and of them, only the cytokine MCP-3 and the immune inhibitory receptor ligand PD-L1 were COVID-19 specific. This fits to the concept that COVID-19 triggers a cascade of immune response followed by a secondary cytokine wave in some patients, which is responsible for the fatal progression [24,25].

In our study, the circulating hormone ADM was the strongest single predictive marker that was associated with COVID-19 mortality and outperformed all other immune markers as well as laboratory parameters in its predictive power (AUROC = 0.87). These findings support further focused evaluation of ADM’s pathophysiological role in organ failure and COVID-19 and its application as powerful diagnostic marker and potentially therapeutic target in COVID-19 patients [29].

Although ADM and other markers had a strong association with clinical outcomes on their own, we developed at a composite marker combining the circulating hormone ADM, the cytokines IL-6 and MCP-3, the apoptosis related receptor TRAIL-2R, and the immune inhibitory receptor ligand PD-L1 in order to further increase the predictive performance.

The combination of several markers to a COVID-19 risk score balances inter- and intra-patient variability due to the individual immune repertoire, comorbidities, and actual therapy, reflected by genetic aspects influencing the circulating proteomes [30]. Potential technical imprecisions can also be adjusted. The robustness of our composite COVID-19 risk PCA analysis is impressively illustrated in the kinetics during hospitalization. With the exception of the apoptosis-related protein TRAIL-R2, which was dynamically increased in those patients with fatal outcome, the other markers displayed a rather steady state. Stable high levels of the composite score were seen in the high-risk patients, despite differences in clinical treatment, e.g., admission to the ICU with or without indication for MV. We believe that refinement, by inclusion and exclusion of markers, and further standardization will increase the clinical utility for a potential diagnostic assay.

### 4.5. What Are Clinical Implications and Do Possible Therapeutic Targets Exist?

A better understanding of the underlying mechanisms resulting in distinct proteome changes and the proteins involved may enable targeted therapeutic approaches. Three of the five key proteins identified in this study in PCA sorting analyses have already been identified as therapeutic targets in COVID-19, and clinical trials are currently ongoing.

Inhibitors of IL-6 and its receptors, such as Tocilizumab, have been approved by the FDA for treating chimeric antigen receptor (CAR)-T-cell mediated cytokine release syndrome (CRS), and have been proposed as a new therapeutic strategy for other types of cytokine wave including sepsis [31]. Indeed, recent studies provided data in favor of improved outcome in critically ill patients with COVID-19 receiving IL-6 antagonists [32,33,34], while other studies produced mixed results [35,36].

ADM is a peptide hormone modulating endothelial function, potent vasodilator, and immune modulator [37], correlating with mortality in septic patients [38]. Like in our study, elevated levels of ADM were also observed in severe COVID-19 cases [39]. The anti-ADM antibody Adrecizumab stabilizes and maintains the endothelial barrier function and is thus supposed to reduce capillary leakage in septic shock, which is also described as a pathomechanism in COVID-19 [40,41]. In a small case series with eight critically ill COVID-19 patients, the administration of Adrecizumab seemed to favor the clinical outcome [42].

PD-1 (CD 279) is a regulator of T cell responses and immunological tolerance [43]. The expression of its cognate ligands PD-L1 (CD 274) and PD-L2 (CD 273) can be increased by various viruses [44]. Elevated blood levels of the soluble PD-L1 isoform seem to inhibit T-cell functions and thus be a marker of T-cell-exhaustion [45].

In our study, PD-L1 was not only one of our composite markers in PCA analyzes predicting clinical outcome but was also significantly higher in COVID-19 patients compared to the symptomatic non-COVID-19 group. This extends current findings of significant difference of soluble PD-L1 levels at hospital admission between mild/moderate and severe/critical COVID-19 cases [45,46].

### 4.6. Can the Multiplexed Proteomics Technique Be Implemented in Clinical Routine as a Diagnostic Method?

The PEA technology has several advantages compared to conventional antibody based assays [20,47] and possibly “omic”-technologies [16]: (1) the technique is ultrasensitive allowing parallel detection of multiple proteins comparable to commercially available singleplex immunoassays; (2) the use of paired antibodies and matching of complementary DNA tags to detect and report each protein at a high specificity; (3) the required plasma volume is minimal, in the range of microliters; and (4) the system is flexible to exclude and include new targets. Moreover, the technical reproducibility is highly indicated by the strong correlation (correlation coefficient 0.972–0.997) of the levels of same targets from different plates.

Although we believe our findings are solid and supported by previous research, the results should be interpreted with caution because of its observational nature. Most associations of markers and outcomes were highly significant, but ultimate confirmation can only be obtained in independent COVID-19 patient cohorts. The same is true for the construction of a risk score based on the combination of prognostic markers, which may imply overfitting. However, recent studies applying also PEA technology identified an overlap of proteins with our results in non-COVID-19 and COVID-19 patients in the ED, when comparing COVID-19 patients with healthy controls, e.g., CXCL10, CXCL11, IFN gamma, MCP-2, MCP-3, and TNF [48], and regarding HGF in hospitalized vs. non-hospitalized patients [46]. Finally, it needs to be stressed that our clinical data were obtained early in the pandemic when clinical evaluation and management of COVID-19 patients, in particular when presented to the ED, was driven by empirical considerations. Since then, optimized management has decreased the morbidity and mortality of COVID-19.

### 4.7. Concluding Remarks

In summary, we demonstrated diverse immune traits that were associated with the pathophysiology and the clinical outcome of COVID-19. As such, increases of pro-inflammatory markers underline disease severity dependent changes in the plasma proteome. The dynamic signatures of markers related to immune response and vascular homeostasis broaden our understanding of the systemic reaction to COVID-19 and will help to optimize biological and clinical assessment of proteins in the context of COVID-19. It is important to note that we employed a high significance level (*p* < 0.001) in our evaluations of proteomics data because we analyzed a high number of analytes in the samples from the different cohorts. This approach was used to avoid detecting random differences in the study population. While many aspects are still enigmatic in the pathogenesis of COVID-19, our study stresses the potential to monitor the suggested mediators of inflammation for informed risk stratification and alternative intervention.

## Figures and Tables

**Table 1:** Selected protein levels analyzed by proximity extension assay (PEA), number of valid measurements, interquartile ranges, and significances (*p*-values) of the non-COVID-19 and the COVID-19 group at the time of admission to the ED. PEA proteome values are selected on the basis of significant differences either between non-COVID-19 patients and COVID-19 patients, or within the COVID-19 patient group regarding significant predictive impact (refer to Table 2). Only *p*-values < 0.001 are depicted in bold.

	Non-COVID-19 Group			COVID-19 Group			
		(N = 44)			(N = 97)		
Parameter	Valid Values	Median Value	Interquartile Ranges	Valid Values	Median Value	Interquartile Ranges	*p*-Value
ADM	44	8.05	6.96, 8.74	95	7.64	7.21, 8.54	0.5725400
CCL23	41	10.43	9.99, 11.58	96	10.41	9.65, 10.99	0.0787430
CTSL1	44	8.73	8.52, 8.99	95	8.62	8.43, 8.91	0.6467800
CXCL10	41	9.88	8.96, 11.07	96	12.69	11.90, 13.34	**0.0000004**
CXCL11	41	10.30	9.90, 11.15	96	12.05	10.75, 12.53	**0.0000039**
CXCL5	41	12.18	11.29, 12.50	96	11.52	10.83, 12.04	**0.0006775**
DCN	44	3.99	3.70, 4.39	95	4.12	3.70, 4.57	0.4066400
Gal9	44	4.76	4.13, 5.11	95	5.70	5.10, 6.58	**0.0000001**
HGF	41	7.72	7.16, 8.19	96	7.45	6.76, 8.15	0.4525200
IFNgamma	41	2.33	2.33, 3.01	96	4.67	2.96, 6.01	**0.0000160**
IL18	44	7.64	7.22, 8.07	95	8.33	7.80, 8.75	**0.0007774**
IL18R1	41	7.35	7.08, 7.56	96	7.92	7.53, 8.45	**0.0000445**
IL27	44	3.20	2.87, 3.69	95	3.36	3.01, 3.90	0.5745600
IL6	44	4.18	2.42, 6.56	95	4.72	3.50, 5.84	0.0639610
KIM1	44	8.21	7.47, 8.81	95	7.86	7.08, 8.79	0.7888000
LIFR	41	4.33	4.04, 4.54	96	4.63	4.35, 4.90	**0.0005687**
LPL	44	7.49	6.99, 8.16	95	7.52	6.94, 8.03	0.8819400
MCP1	41	11.72	11.33, 12.14	96	12.33	11.65, 12.82	0.0240940
MCP2	41	8.81	8.37, 9.16	96	10.69	9.81, 11.58	**0.00000001**
MCP3	41	3.98	3.43, 4.95	96	5.43	4.55, 6.77	**0.0003295**
MERTK	44	6.55	6.23, 6.83	95	6.96	6.56, 7.26	**0.0007555**
MMP1	41	13.25	12.28, 14.66	96	13.00	11.86, 13.89	**0.0007128**
MMP12	44	6.41	5.64, 7.37	95	5.80	4.97, 6.93	0.1359200
OPG	41	9.35	8.83, 9.99	96	9.11	8.65, 9.78	0.1229900
PDL1	41	4.39	4.06, 4.64	96	5.14	4.74, 5.61	**0.0000002**
TNF	41	1.59	1.21, 2.03	96	2.15	1.80, 2.63	**0.0002014**
TNFRSF10A	44	2.52	1.97, 2.96	95	2.49	2.09, 3.23	0.8851000
TRAIL-R2	44	6.33	5.76, 7.17	95	6.05	5.48, 7.01	0.2625300

**Table 2:** Levels of selected circulating proteins and standard of care parameter (SoC) levels, interquartile ranges and significances (*p*-values) of the COVID-19 group with regard to the clinical endpoints hospitalization, intensive care unit (ICU) treatment, indication for mechanical ventilation (MV), thromboembolic event (TE), and death. Only analytes with significant discrimination either between non-COVID-19 and COVID-19 patients, or within hospitalized and non-hospitalized COVID-19 patients are shown. Only *p*-values < 0.001 are depicted in bold.

	Median and Interquartile Ranges for Non-Outcomes				Median and Interquartile Ranges for Outcomes				*p*-Values Non-Outcome vs. Outcome			
	ICU	MV	TE	Death	ICU	MV	TE	Death	ICU	MV	TE	Death
	29 (54.72%)	37 (69.81%)	47 (88.68%)	46 (86.79%)	24 (45.28%)	16 (30.19%)	6 (11.32%)	7 (13.21%)				
SoC analytes												
CRP	36.5 (14.6–62.6)	35.3 (15.8–66.1)	44.4 (16.5–89.7)	45.3 (16.4–106)	110 (25.8–155)	140 (59.8–216)	187 (39.4–265)	53.7 (22.8–129)	0.020	0.011	0.175	0.637
PCT	0.10 (0.08–0.24)	0.10 (0.08–0.24)	0.10 (0.08–0.25)	0.11 (0.08–0.26)	0.17 (0.10–0.30)	0.20 (0.10–0.62)	0.47 (0.20–0.78)	0.11 (0.10–0.25)	0.241	0.160	0.082	0.897
WBC	6.35 (5.33–8.51)	6.62 (5.27–8.63)	6.35 (5.26–8.62)	7.12 (5.29–10.3)	7.49 (5.13–10.9)	7.64 (5.33–12.0)	9.35 (9.12–10.5)	5.65 (5.05–7.35)	0.372	0.416	0.106	0.325
LDH	300 (266–399)	295 (263–438)	322 (271–504)	340 (269–563)	509 (439–684)	509 (453–718)	461 (447–589)	432 (300–460)	0.014	0.006	0.196	0.978
PEA analytes												
ADM	8.37 (7.40–8.78)	8.30 (7.38–8.66)	8.37 (7.49–8.80)	8.28 (7.44–8.65)	8.41 (7.54–8.82)	8.64 (8.16–8.90)	8.50 (7.77–8.75)	8.98 (8.76–9.12)	0.1207000	0.0472670	0.5869700	**0.0000932**
CCL23	10.5 (9.92–10.9)	10.5 (9.92–11.1)	10.8 (10.2–11.2)	10.6 (10.1–11.1)	11.1 (10.6–11.6)	11.2 (10.7–11.6)	10.7 (9.81–11.1)	11.3 (10.8–11.6)	**0.0006146**	0.0018489	0.8367700	0.0551560
CTSL1	8.72 (8.52–9.03)	8.75 (8.52–9.03)	8.75 (8.53–9.04)	8.80 (8.52–9.10)	8.86 (8.68–9.18)	8.86 (8.70–9.26)	9.17 (8.88–9.18)	8.78 (8.67–8.99)	0.0085455	0.0472670	0.0173740	0.3078300
CXCL10	12.6 (12.0–13.3)	12.7 (12.0–13.3)	12.9 (12.0–13.3)	12.7 (11.2–13.3)	13.1 (11.9–13.5)	13.1 (12.4–13.6)	13.6 (10.7–13.8)	13.7 (13.2–13.8)	0.0669700	0.1257600	0.0160730	0.0024786
CXCL11	11.8 (10.9–12.4)	11.9 (10.8–12.5)	12.2 (10.9–12.5)	12.0 (10.7–12.5)	12.5 (11.3–12.7)	12.4 (12.1–12.6)	12.6 (11.2–12.6)	12.6 (12.6–12.6)	0.1027300	0.1931000	0.3580900	0.0109360
CXCL5	11.6 (11.2–12.2)	11.5 (11.1–12.0)	11.5 (11.1–12.0)	11.4 (11.1–12.0)	11.3 (10.8–12.0)	11.3 (11.1–12.2)	11.2 (11.1–12.0)	11.7 (10.6–12.3)	0.6541900	0.2407300	0.1392900	0.0403690
DCN	4.35 (3.84–4.77)	4.22 (3.83–4.71)	4.35 (3.84–4.82)	4.30 (3.84–4.70)	4.41 (4.01–4.75)	4.64 (4.37–5.10)	4.60 (4.42–4.72)	5.31 (4.56–5.62)	0.0475360	**0.0009899**	0.0154320	0.0074615
Gal9	5.90 (5.10–6.77)	5.90 (5.08–6.77)	5.93 (5.14–6.72)	5.78 (5.08–6.72)	5.99 (5.21–6.82)	6.05 (5.59–6.82)	6.27 (4.59–7.00)	6.60 (6.05–6.86)	0.7322500	0.5908400	0.1927500	0.0442300
HGF	7.54 (7.25–8.20)	7.54 (7.31–8.20)	7.64 (7.32–8.23)	7.56 (7.32–8.36)	8.00 (7.44–8.59)	8.16 (7.59–8.59)	8.25 (7.71–8.41)	8.16 (7.83–8.27)	0.0354240	0.0452030	0.3725600	0.1236400
IFNgamma	4.59 (2.60–6.00)	4.63 (2.79–6.04)	4.80 (3.19–6.03)	4.67 (3.15–6.03)	4.95 (3.64–5.63)	4.89 (3.20–5.21)	3.50 (2.40–4.86)	4.53 (3.09–5.31)	0.1746500	0.6409200	0.8324600	0.8324600
IL18	8.42 (8.09–8.71)	8.36 (8.05–8.67)	8.44 (8.14–8.86)	8.43 (8.09–8.90)	8.51 (8.16–9.41)	8.64 (8.27–9.56)	8.42 (7.60–9.17)	8.66 (8.26–9.03)	0.3469200	0.0557820	0.3616600	0.3725600
IL18R1	8.05 (7.60–8.42)	8.05 (7.59–8.53)	8.09 (7.61–8.49)	7.98 (7.61–8.47)	8.35 (7.70–8.62)	8.23 (7.84–8.55)	8.12 (7.65–8.59)	8.55 (8.30–8.64)	0.4282800	0.2964400	0.8367700	0.1236400
IL27	3.70 (3.27–4.15)	3.62 (3.07–3.98)	3.68 (3.09–4.11)	3.62 (3.04–3.96)	3.67 (3.06–4.04)	3.90 (3.45–4.35)	3.68 (3.43–4.13)	4.42 (4.05–4.49)	0.3287600	0.0472670	0.4544500	0.0074615
IL6	4.84 (4.33–5.55)	4.86 (4.33–6.01)	5.27 (4.66–6.61)	5.05 (4.40–6.26)	6.43 (5.37–6.96)	6.74 (5.60–7.31)	5.84 (4.36–6.54)	6.62 (5.55–6.80)	**0.0000044**	**0.0000002**	0.3580900	0.0319650
KIM1	8.51 (8.13–9.53)	8.38 (7.75–9.07)	8.41 (7.71–9.08)	8.37 (7.69–9.09)	8.06 (7.62–8.85)	8.68 (7.78–9.32)	8.72 (7.96–9.39)	9.01 (8.68–9.35)	0.1353200	0.1374300	0.8455500	0.0442300
LIFR	4.66 (4.42–4.94)	4.73 (4.49–4.92)	4.80 (4.50–4.93)	4.76 (4.50–4.92)	4.84 (4.66–4.93)	4.84 (4.67–5.10)	4.93 (4.66–4.99)	5.07 (4.86–5.38)	0.0197430	0.0853480	0.3725600	0.0551560
LPL	7.72 (6.81–8.44)	7.72 (7.06–8.29)	7.66 (7.09–8.35)	7.62 (7.07–8.10)	7.65 (7.41–8.14)	7.65 (7.51–8.67)	7.78 (7.54–8.37)	8.73 (8.10–9.06)	0.4779700	0.1412000	0.5225300	**0.0001652**
MCP1	12.4 (11.8–12.8)	12.3 (11.8–12.8)	12.5 (11.8–13.0)	12.5 (11.7–13.0)	13.0 (12.1–13.5)	13.3 (12.6–13.7)	13.4 (12.0–13.8)	13.5 (13.0–13.7)	**0.0000581**	**0.0000419**	0.0545020	0.0024786
MCP2	10.5 (9.34–11.2)	10.8 (9.24–11.7)	10.8 (9.48–11.5)	10.8 (9.26–11.5)	11.1 (9.60–12.0)	11.0 (9.95–12.0)	12.0 (9.91–12.0)	12.2 (11.3–12.4)	0.0934230	0.4824300	0.0545020	0.0024786
MCP3	5.42 (4.70–6.70)	5.59 (4.72–6.74)	6.11 (4.76–7.09)	6.11 (4.76–7.16)	6.93 (5.98–7.87)	7.43 (6.39–8.22)	7.66 (5.99–8.11)	7.29 (5.63–8.11)	**0.0001310**	**0.0000231**	0.0024786	0.0571620
MERTK	6.96 (6.79–7.39)	6.96 (6.79–7.40)	7.02 (6.82–7.37)	6.97 (6.80–7.37)	7.10 (6.89–7.43)	7.16 (6.95–7.43)	7.32 (6.72–7.79)	7.25 (7.04–7.52)	0.1706600	0.1503800	0.0580550	0.2731700
MMP1	13.6 (12.8–14.1)	13.5 (12.5–14.0)	13.5 (12.6–14.2)	13.4 (12.5–14.0)	13.5 (12.5–14.3)	14.1 (12.6–14.5)	14.0 (12.9–14.3)	14.3 (14.1–14.5)	0.1102800	0.0158300	0.5116700	0.0551560
MMP12	6.34 (5.38–7.30)	5.98 (5.27–7.14)	6.10 (5.28–7.30)	5.84 (5.23–6.98)	5.87 (5.22–7.21)	6.36 (5.24–7.54)	5.75 (5.34–6.70)	7.32 (6.92–7.99)	0.2720700	0.2977100	0.8455500	0.1061200
OPG	9.54 (8.97–10.1)	9.50 (8.97–9.92)	9.52 (8.97–10.1)	9.55 (8.97–9.93)	9.59 (9.02–10.1)	9.82 (9.40–10.2)	9.72 (9.59–10.0)	10.1 (9.69–10.2)	0.0499440	0.0242520	0.0109360	0.0551560
PDL1	5.04 (4.81–5.46)	5.19 (4.81–5.61)	5.34 (4.90–5.73)	5.29 (4.82–5.72)	5.49 (5.17–5.82)	5.49 (5.29–5.80)	5.25 (4.59–5.68)	5.49 (5.33–5.77)	**0.0006113**	0.0528220	0.8324600	0.1714200
TNF	2.49 (2.02–2.87)	2.33 (1.89–2.79)	2.32 (1.92–2.77)	2.18 (1.83–2.65)	2.05 (1.85–2.41)	2.23 (1.92–2.63)	2.31 (1.76–2.46)	2.72 (2.63–3.10)	0.0669310	0.4650900	0.9491500	0.1859300
TNFRSF10A	2.74 (2.40–3.25)	2.65 (2.40–3.27)	2.87 (2.44–3.43)	2.73 (2.41–3.41)	3.16 (2.50–3.61)	3.30 (2.68–3.72)	3.02 (2.46–3.53)	3.25 (2.91–3.68)	0.0168280	0.0074506	0.5225300	0.1002400
TRAILR2	6.40 (5.83–7.09)	6.40 (5.77–7.08)	6.75 (5.91–7.22)	6.52 (5.85–7.09)	7.01 (6.46–7.35)	7.16 (6.71–7.63)	7.06 (6.41–7.16)	7.18 (7.07–7.85)	**0.0004393**	**0.0000823**	0.5869700	0.0125550

## Data Availability

The data presented in this study are available on request from the corresponding author.

## Supplementary Materials

The following are available online at https://www.mdpi.com/article/10.3390/v13122456/s1, Supplemental Figure S1: Correlation matrix between the selected markers and the standard of care infection markers WBC, CRP, and PCT. Blank cells were statistically insignificant. Supplemental Table S1: Baseline demographics of patients included in this study. Supplemental Table S2: Clinical chemistry, hematology, and hemostaseology of patient groups at the time of admission to the emergency department. Supplemental Table S3: Multiplexed protein analyses. Supplemental Table S4: AUROCs and clinical outcome.

## Abbreviations

AUROC = area under the receiver operating characteristic; COVID-19 = coronavirus disease 2019; CRP = C-reactive protein; ED = emergency department; eGFR = estimated glomerular filtration rate; ICU = intensive care Unit; IL = interleukin; LDH = lactate dehydrogenase; MV = mechanical ventilation; NPX = normalized protein expression; PEA = proximity extension assay; PCA = principal component analysis; PCR = polymerase chain reaction; PCT = procalcitonin; SARS-CoV-2 = severe acute respiratory syndrome coronavirus 2; TE = thromboembolic event; WBC = white blood cell count.
