# Supplementary material for: Plasma Proteome Fingerprints Reveal Distinctiveness and Clinical Outcome of SARS-CoV-2 Infection
Source: Viruses. 2021 Dec 7;13(12):2456. doi: 10.3390/v13122456 (PMC8706135; doi:10.3390/v13122456)
Supplement: Supplementary file 1 [file viruses-13-02456-s001.zip › viruses-1444054-supplementary/Table S1 Supplemental.pdf]

Supplemental Table S1.

	Reference cohort	Non-COVID-19	Non-hospitalized COVID-19	Hospitalized COVID-19			
	(N = 20)	(N = 44)	(N = 44)	(N = 53)	p-value	p-value	p-value
	Group 1	Group 2	Group 3	Group 4	1 vs. 2	2 vs. 3+4	3 vs. 4
**Demographics**							
Age [years]	55.6 (44.9, 67.6)	63.1 (50.1, 77.5)	37.2 (27.7, 52.7)	71.2 (54.1, 79.2)	0.143	**0.040**	**<0.001**
Female	12 (60%)	23 (52%)	19 (43%)	27 (51%)	0.600	0.716	0.541
**Comorbidity**							
CKD	1/19 (5%)	9 (20%)	3 (7%)	11 (21%)	0.258	0.461	0.080
CHD	1/19 (5%)	5 (11%)	4 (9%)	11 (21%)	0.658	0.610	0.160
Hypertension	5/19 (26%)	18 (41%)	8 (18%)	28 (53%)	0.394	0.711	**<0.001**
T2DM	2/19 (11%)	3 (7%)	6 (14%)	13 (25%)	0.633	0.078	0.207
COPD	0/19 (0%)	3 (7%)	0/43 (0%)	6 (11%)	0.547	1.000	**0.031**
Asthma	1/19 (5%)	1 (2%)	2 (5%)	9 (17%)	0.516	0.104	0.104
MD	1/19 (5%)	7/43 (16%)	1 (2%)	1 (2%)	0.416	**0.004**	1.000
OAC	1/19 (5%)	9/43 (21%)	0/43 (0%)	10 (19%)	0.155	0.112	**0.002**
**Treatment**							
Length of stay	1.0 (1.0, 4.2)	3.0 (1.0, 9.0)	1.0 (1.0, 1.0)	12.0 (8.0, 20.0)	0.204	0.129	**<0.001**

CKD = chronic kidney disease; CHD=coronary heart disease; T2DM=type 2 diabetes mellitus; COPD=chronic obstructive pulmonary disease; MD=malignant disease; OAC=oral anticoagulation.
