# Supplementary material for: Plasma Proteome Fingerprints Reveal Distinctiveness and Clinical Outcome of SARS-CoV-2 Infection
Source: Viruses. 2021 Dec 7;13(12):2456. doi: 10.3390/v13122456 (PMC8706135; doi:10.3390/v13122456)
Supplement: Supplementary file 1 [file viruses-13-02456-s001.zip › viruses-1444054-supplementary/Table S2 Supplemental.pdf]

	Non-COVID-19 n=44			Non-hospitalized COVID-19 n=44			Hospitalized COVID-19 n=53			Non-COVID-19 vs. COVID-19	Inpatient vs. Outpatient
	n	median	IQR	n	median	IQR	n	median	IQR		
**Clinical Chemistry**											
NA	5	139	132.00, 141.00	4	137	136.25, 137.50	10	138	137.25, 139.75	1.000	0.317
K	5	4.6	3.70, 36.00	4	4.35	4.05, 13.88	10	4.6	4.15, 27.50	0.817	0.831
CA	1	2.27	2.27, 2.27	3	2.26	2.23, 2.33	2	2.38	2.34, 2.41	1.000	0.400
CK	26	86.5	54.50, 123.50	14	82.5	65.00, 159.75	27	133	68.00, 218.50	0.080	0.196
Creatinine	44	0.92	0.79, 1.16	41	0.84	0.68, 1.03	50	1.02	0.83, 1.35	0.732	0.005
eGFR	44	74.5	53.00, 91.00	39	91	85.00, 91.00	49	63	38.00, 84.00	0.594	**<0.001**
AST	18	28.5	21.50, 35.50	9	30	25.00, 42.00	25	38	30.00, 57.00	0.023	0.191
ALT	29	20	18.00, 26.00	31	24	15.50, 43.00	41	32	20.00, 49.00	0.010	0.097
GGT	28	25	19.75, 70.50	27	31	21.50, 78.50	38	53	34.00, 98.75	0.076	0.052
LDH	23	288	251.50, 392.50	26	262	233.25, 323.50	29	342	272.00, 518.00	0.504	**<0.001**
Bilirubin	28	0.37	0.29, 0.66	24	0.32	0.25, 0.44	36	0.52	0.31, 0.73	0.989	0.025
TNT	13	4	3.00, 20.00	9	5	3.00, 9.00	12	34	16.00, 111.00	0.218	**<0.001**
FERR	0	NA	NA	9	207.9	135.60, 627.00	7	2305	444.65, 3220.45	NA	0.114
CRP	42	9.9	1.57, 64.90	43	11.2	4.80, 30.60	49	46.2	16.20, 120.80	0.175	**<0.001**
PCT	27	0.08	0.04, 0.15	22	0.05	0.04, 0.10	32	0.11	0.08, 0.25	0.345	**<0.001**
**Coagulation**											
INR	38	1.08	1.00, 1.18	35	1.05	1.00, 1.10	49	1.09	1.02, 1.27	0.916	0.060
aPTT	38	34	31.13, 36.30	35	35.2	33.10, 37.75	49	34.5	32.90, 38.30	0.176	0.713
**Hemostaseology**											
WBC	43	9.33	7.56, 11.68	44	5.62	4.15, 6.59	53	6.68	5.27, 9.36	**<0.001**	0.016
Hb	43	13	11.60, 14.00	44	13.25	12.10, 14.33	53	13.4	11.60, 14.10	0.629	0.882
HCT	43	0.38	0.35, 0.41	44	0.39	0.35, 0.42	53	0.39	0.34, 0.42	0.948	0.714
RBC	43	4.5	4.00, 4.80	44	4.75	4.38, 5.10	53	4.5	4.00, 5.10	0.250	0.157
MCV	43	86	83.00, 90.00	44	83	79.75, 85.25	53	86	81.00, 89.00	0.048	0.006
MCH	43	29.2	28.30, 31.00	44	28.5	26.98, 29.52	53	29.3	28.40, 30.70	0.167	0.008
MCHC	43	34	33.30, 34.90	44	34	33.27, 34.73	53	34.1	33.30, 35.00	0.578	0.609
RDW	42	13.9	12.53, 15.30	44	12.8	12.47, 13.53	53	13.4	12.90, 14.70	0.156	0.007
PLT	43	263	210.50, 318.50	44	206	172.75, 242.25	53	227	180.00, 271.00	0.001	0.120
MPV	42	10.15	9.53, 10.70	43	10.3	9.95, 11.10	52	10.3	9.80, 11.00	0.108	0.742

Supplemental Table S2

Diagnostics were performed at presentation to the ED as standard of care. Abbreviations: white blood cells (WBC), hemoglobin (HB), hematocrit (HCT) red blood cells (RBC), mean corpuscular volume (MCV), mean corpuscular hemoglobin (MCH), mean corpuscular hemoglobin concentration (MCHC), red distribution width (RDW), platelets (PLT), mean platelet volume (MPV), sodium (NA), potassium (K), Calcium (CA), creatine kinase (CK), calculated estimated glomerular filtration rate (eGFR), aspartate aminotransferase (AST), alanine aminotransferase (ALT), gamma-glutamyltransferase (GGT), lactate dehydrogenase (LDH), total bilirubin, troponin T (TNT), C-reactive protein (CRP), procalcitonin (PCT), activated partial thromboplastin time (aPTT, coagulometry), prothrombin time/international normalized ratio (INR, coagulometry).
