# Supplementary figures and images for: Plasma Proteome Fingerprints Reveal Distinctiveness and Clinical Outcome of SARS-CoV-2 Infection
Source: Viruses. 2021 Dec 7;13(12):2456. doi: 10.3390/v13122456 (PMC8706135; doi:10.3390/v13122456)

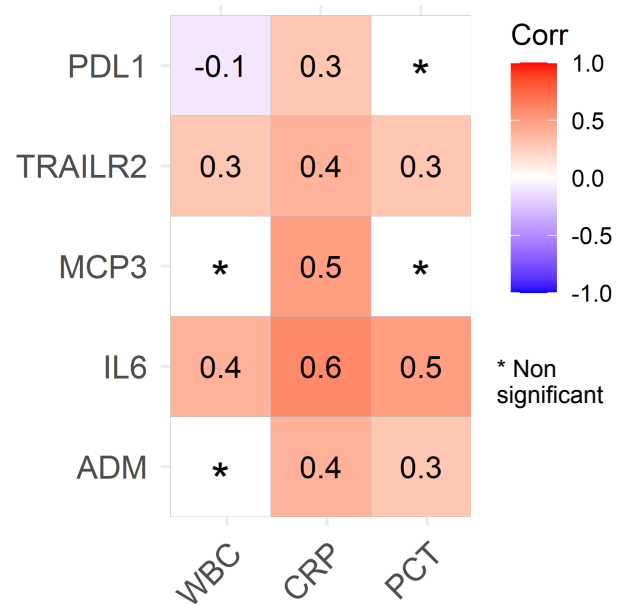

**Supplemental Figure S1**

Supplement: Supplementary file 1 [file viruses-13-02456-s001.zip › viruses-1444054-supplementary/Figure S1 Supplemental.pdf]
